# Associations of IGF-1, vitamin D, and bone minerals with short stature in children aged 24–59 months

**DOI:** 10.3389/fendo.2026.1759569

**Published:** 2026-06-12

**Authors:** Novina Novina, Aman Bhakti Pulungan, Michael Hermanussen, Agustini Utari, Yoyos Dias Ismiarto, Meita Dhamayanti, Eddy Fadlyana, Yudi Mulyana Hidayat, Raden Tina Dewi Judistiani, Vitriana Biben, Rovina Ruslami, Elrika Anastasia, Yola Herniati Herjanyo, Nadia Pramudani, Budi Setiabudiawan

**Affiliations:** 1 Department of Child Health, Faculty of Medicine, Universitas Padjadjaran, Bandung, Indonesia; 2 Department of Child Health, Faculty of Medicine, Universitas Indonesia, Jakarta, Indonesia; 3 Department of Child Health, Kiel University, Kiel, Germany; 4 Department of Child Health, Faculty of Medicine, Universitas Diponegoro, Semarang, Indonesia; 5 Department of Orthopedic and Traumatology, Faculty of Medicine, Universitas Padjadjaran, Bandung, Indonesia; 6 Department of Obstetrics and Gynecology, Faculty of Medicine, Universitas Padjadjaran, Bandung, Indonesia; 7 Department of Public Health, Faculty of Medicine, Universitas Padjadjaran, Bandung, Indonesia; 8 Department of Physical Medicine and Rehabilitation, Faculty of Medicine, Universitas Padjadjaran, Bandung, Indonesia; 9 Department of Biomedical Sciences, Faculty of Medicine, Universitas Padjadjaran, Bandung, Indonesia; 10 Department of Internal Medicine, Faculty of Medicine, Universitas Padjadjaran, Bandung, Indonesia

**Keywords:** calcium, children, IGF-1, magnesium, phosphorus, short stature, underweight children, vitamin D

## Abstract

**Background:**

Child growth results from the interaction of genetic, endocrine, nutritional, and environmental factors. Short stature is defined as a height-for-age Z-score below −2 standard deviations, according to the WHO Child Growth Standards 2006. Linear bone growth occurs through endochondral ossification, which is influenced by insulin-like growth factor-1 (IGF-1), 25-hydroxyvitamin D [vitamin 25(OH) D], calcium (Ca), phosphorus (Ph), and magnesium (Mg). This study compared these biomarkers across growth phenotypes and examined their associations with short stature in children aged 24–59 months.

**Methods:**

This cross-sectional analytical study used registry data and stored biological specimens (SBS) from 225 children aged 24–59 months residing in Bandung District, Indonesia (84 underweight children with short stature, 70 normal-weight children with short stature, and 71 children with normal weight and stature), collected between May and August 2021. SBS were preserved at -80 °C. Appropriate statistical tests, *post-hoc* analyses, and multivariable logistic regression were performed.

**Results:**

Significant differences in IGF-1, 25(OH)D, calcium, phosphorus, and magnesium were observed across groups (all p < 0.05). *Post-hoc* analysis indicated that these differences were confined to comparisons between the underweight short stature group and the normal-weight normal stature group, with no significant differences involving the normal-weight short stature group. Multivariable analysis further showed that lower levels of IGF-1, calcium, phosphorus, and magnesium were independently associated with short stature, with these associations largely attributable to the underweight subgroup, while remaining non-significant among children with normal-weight short stature.

**Conclusion:**

Lower levels of IGF-1, calcium, phosphorus, and magnesium are associated with short stature in children aged 24–59 months, predominantly among those with concurrent undernutrition. In contrast, these associations were not evident in children with normal-weight short stature, underscoring the heterogeneous nature of short stature.

## 1 Introduction

The Sustainable Development Goals (SDGs) aim to eliminate poverty and hunger while improving the nutritional status of mothers and children. One of the key targets of SDG 2 is a 40% reduction in global stunting prevalence by 2025 (1). In Indonesia, stunting remains a major public health challenge. Between 2005 and 2017, Indonesia had the third-highest prevalence of stunting in Southeast Asia, at 36.4%. More recent national data indicate that the prevalence declined to 19.8% in 2025, achieving the national target of below 20% (2, 3).

Short stature is a clinical condition defined as a length-for-age Z-score (LAZ) or height-for-age Z-score (HAZ) below −2 standard deviations (SD) based on the WHO Child Growth Standards 2006. It reflects an individual child’s growth status and may arise from normal variants, genetic factors, endocrine disorders, or chronic systemic diseases. In contrast, stunting is a population-level indicator of chronic undernutrition, also defined by HAZ < −2 SD, typically originating during the first 1,000 days of life. Stunting is associated with prolonged exposure to inadequate nutrition, recurrent infections, poor maternal health, micronutrient deficiencies, low socioeconomic status, and adverse environmental conditions (1, 4–7). Nutritional status is commonly assessed using weight-for-height or BMI-for-age based on WHO standards for children under five years and CDC 2000 charts for older children (5, 8).

Growth in children is a complex process influenced by interactions among genetic, endocrine, nutritional, and environmental factors. Linear growth is assessed by increases in height resulting from endochondral ossification at the growth plate (9–11). This process is tightly regulated by endocrine factors, particularly growth hormone (GH) and vitamin D. GH stimulates hepatic production of insulin-like growth factor-1 (IGF-1), which promotes chondrocyte proliferation and hypertrophy at the epiphyseal growth plate, thereby supporting longitudinal bone growth. Vitamin D contributes to bone formation and remodeling by maintaining calcium and phosphorus homeostasis through intestinal absorption, bone turnover, and renal regulation. Optimal bone growth also requires coordinated regulation of parathyroid hormone (PTH) and calcitonin. In addition, magnesium plays an essential role in vitamin D activation and PTH secretion; deficiencies in these minerals may impair growth plate function and are commonly observed in pediatric bone disorders such as rickets (12, 13).

Despite the biological importance of these biomarkers, studies simultaneously evaluating endocrine and mineral parameters in children with growth impairment remain limited, particularly in Indonesia. Previous studies have reported lower IGF-1 levels and micronutrient deficiencies in short stature children, although findings vary across populations. For example, Senudin et al. (14) reported significantly lower serum IGF-1 levels and reduced mineral intake among Indonesian children with stunting (14). In addition, a meta-analysis demonstrated an increased risk of vitamin D deficiency in undernourished children (15), supported by regional studies showing lower 25(OH)D levels in short stature populations (16). However, existing studies have largely focused on short stature populations without distinguishing between different growth phenotypes. Consequently, it remains uncertain whether children with short stature but adequate nutritional status share similar biochemical profiles. Clarifying this distinction is essential to better understand the heterogeneity of short stature and to avoid attributing growth impairment exclusively to nutritional factors.

Therefore, this study aimed to compare serum levels of IGF-1, 25(OH)D, calcium, phosphorus, and magnesium among children with underweight short stature, normal-weight short stature, and normal-weight normal stature, and to examine their associations with short stature in children aged 24–59 months. We hypothesized that these groups would exhibit distinct biochemical profiles.

## 2 Materials and methods

### 2.1 Study design

This cross-sectional analytical study utilized registry data and stored biological specimens (SBS) derived from a parent study entitled *“The Relationship between Vitamin D and Its Contributing Factors with the Incidence and Comorbidity of Stunting in Children Aged 0–59 Months in Bandung Regency,”* conducted by the Immunology Study Center, Faculty of Medicine, Universitas Padjadjaran, between 2019 and 2020. The parent study primarily included children with stunting and/or underweight, as well as children with normal stature as a comparison group. The blood samples used in the present study were previously collected during routine health assessments in the parent study and were not newly obtained for this analysis. All samples were stored for future research use with prior informed consent from parents or legal guardians.

This study was approved by the Health Research Ethics Committee of Universitas Padjadjaran (Ethical Exemption No. 375/UN6.KEP/EC/2021) and was conducted in accordance with the Declaration of Helsinki.

### 2.2 Study subjects and sampling

The target population of this study was children aged 24–59 months residing in Bandung Regency. The accessible population consisted of children enrolled in the parent study who met the eligibility criteria, had adequate stored serum samples for biochemical analysis, and whose parents or legal guardians had provided informed consent for future research use. No *a priori* sample size calculation was performed, as total sampling was applied using all available SBS from the parent study that met the eligibility criteria. Sampling in the parent study was conducted using a multistage approach combining systematic random sampling and purposive sampling. Initially, 11 districts were randomly selected from 31 districts in Bandung Regency. Subsequently, 39 villages were purposively selected based on accessibility and data availability. From these villages, 29 primary healthcare centers were randomly selected, followed by purposive selection of 12 centers that met logistical and accessibility criteria.

A total of 240 stored biological specimens were initially available from the parent study. Of these, 15 samples were excluded due to insufficient serum volume or incomplete data, resulting in 225 children included in the final analysis.

The inclusion criteria were:

1. Children aged 24–59 months residing in Bandung Regency.
2. Availability of sufficient stored serum volume for biochemical analysis.
3. The exclusion criteria were:
4. Damaged or contaminated blood samples unsuitable for analysis.
5. Incomplete clinical or anthropometric data.
6. History of major congenital anomalies, syndromic conditions, or malignancy.
7. Known chronic diseases affecting growth include anemia, hepatobiliary disease, kidney disease, or bone disorders.
8. Diagnosed malabsorption syndromes, such as celiac disease, or metabolic disorders.
9. History of epilepsy or antiepileptic drug use.

### 2.3 Anthropometric measurements and biochemical analysis

**Figure 1:**
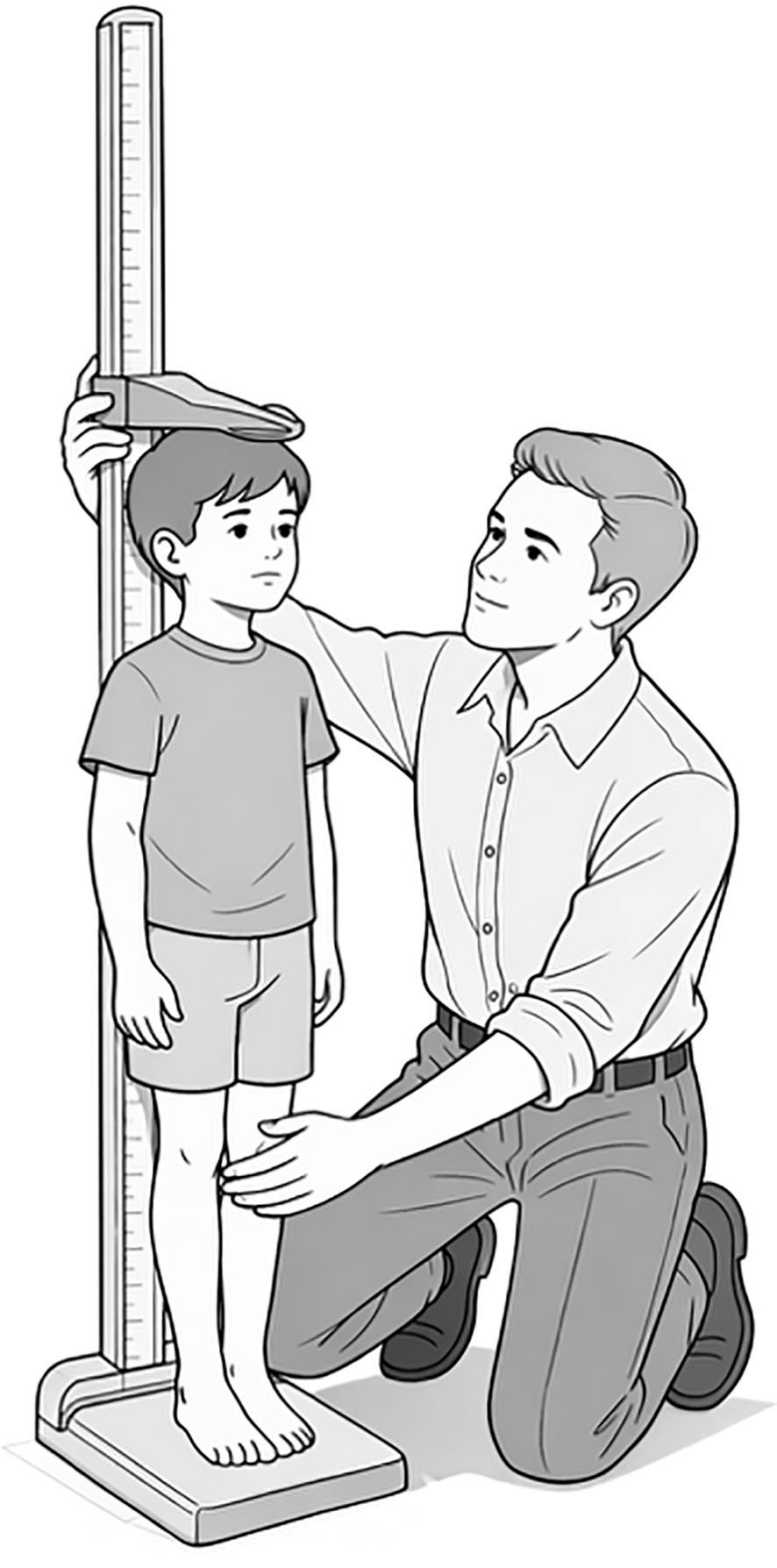
Procedure for measuring the height of children aged ≥ 2 years using a calibrated stadiometer.

Anthropometric data were collected by trained pediatricians. Height measurements for children aged ≥2 years were obtained using a calibrated GEA stadiometer. Measurements were performed with the child standing upright in the Frankfort plane, aligned with the stadiometer, with a measurement precision of 0.1 cm (17, 18). The standardized anthropometric assessment procedures applied across the three study groups are illustrated in **Figure 1**. Data on birth weight and birth length were obtained from parents through structured interviews conducted by trained research assistants. Based on anthropometric classification using WHO Child Growth Standards, the children were categorized into three groups: underweight short stature (WAZ < −2 SD and HAZ < −2 SD and), normal-weight short stature (−2 SD ≤ WAZ ≤ 2 SD and HAZ < −2 SD), and normal-weight normal stature (−2 SD ≤ WAZ ≤ 2 SD and −2 SD ≤ HAZ ≤ 2 SD).

Venous blood samples (5 mL) were originally collected at the time of participant recruitment in the parent study as part of standardized clinical procedures. The present study utilized these stored biological specimens for secondary analysis, and no additional blood collection was performed. Blood samples were transported to the Clinical Pathology Laboratory of Dr. Hasan Sadikin General Hospital, Bandung, for processing. Serum was separated and stored at −80 °C in aliquots until biochemical analysis.

Serum IGF-1 levels were measured using an enzyme-linked immunosorbent assay (ELISA) with Elabscience^®^ kits (19). Age- and sex-specific reference ranges were applied (20). Serum 25-hydroxyvitamin D [vitamin 25(OH)D] levels were measured using an ELISA kit from Euroimmun^®^ (21). Vitamin D status was classified as deficiency (≤20 ng/mL), insufficiency (21–29 ng/mL), and sufficiency (30–100 ng/mL) (22).

Serum calcium levels were measured using the direct ion-selective electrode (ISE) method, with a reference range of 4.8–5.2 mg/dL for children aged 24–59 months (23). Phosphorus levels were determined using a modified phosphomolybdate method (Fiske and Subbarow) with spectrophotometric analysis (24), with a reference range of 4.0–7.0 mg/dL (25). Magnesium levels were measured using a modified complexometric method with methyl thymol blue (MTB) (24), with a reference range of 1.8–2.4 mg/dL (26).

All biochemical analyses were performed at the Clinical Pathology Laboratory of Dr. Hasan Sadikin General Hospital.

### 2.4 Statistical analysis

All study data were recorded on research forms, tabulated, and analyzed using IBM^®^ SPSS^®^ statistic software version 26. Data normality was assessed using the Kolmogorov–Smirnov test prior to analysis. Bivariate comparisons were performed using the independent *t*-test or Mann–Whitney U test for two-group comparisons, and one-way ANOVA or the Kruskal–Wallis test for comparisons among three groups, as appropriate. *Post-hoc* analyses were performed using Tukey’s HSD test for normally distributed data and Dunn’s test with Bonferroni correction for non-parametric data. The chi-square test was used for categorical variables. Variables with a p-value <0.25 in bivariate analysis were included as candidates into the multivariable logistic regression model. Multivariable logistic regression analysis with the Wald test was then performed to evaluate the associations between biomarker levels (IGF-1, vitamin 25(OH)D, calcium, phosphorus, and magnesium) and the occurrence of short stature. A p-value <0.05 was considered statistically significant.

## 3 Result

A total of 225 children aged 24–59 months were included, comprising 154 children with short stature and 71 with normal stature. Comparisons of baseline characteristics between these groups are presented in **Table 1**.

**Table 1:** Basic characteristics of subjects.

Characteristics		Short stature	Normal stature	P value
		HAZ <-2 SD (n=154)	2SD ≤ HAZ ≥- 2SD (n=71)	
Age (month) mean (SD)		44.6 (10.02)	39.75 (9.73)	0.001^*^
Weight/Age, n (%)	>2 SD	0	0	0.001^*^
	2 SD ≤ Weight/Age *≥*- 2SD	70 (45.5)	71 (100)	
	<-2SD	84 (54.5)	0	
BMI/Age, n (%)	>2SD	0	0	0.171^*^
	2 SD ≤ BMI/age ≥- 2SD	150 (97.4)	71 (100)	
	<-2SD	4 (2.59)	0	
Gender, n (%)				0.723^†^
Male		72 (46.8)	35 (49.3)	
Female		82 (53.2)	36 (50.7)	
Birth weight (g) mean (SD)		2,773.08 (479.31)	2,955 (433.25)	0.022^*^
Birth length (cm) mean (SD)		47.85 (2.76)	48.92 (2.08)	0.015^*^
Exclusive breastfeeding, n (%)				<0.001^†^
Yes		137 (89)	45 (63.4)	
No		17 (11)	26 (36.6)	
Immunization administration, n (%)				0.652^*^
Complete		131 (85.1)	62 (87.3)	
Incomplete		23 (14.9)	9 (12.7)	
Father				
Age (year) mean (SD)		31.17 (6.5)	34.35 (7.92)	0.001^*^
Height (cm) mean (SD)		160.44 (6.7)	163.31 (6.79)	0.002^*^
Education, n (%)				0.067^†^
Elementary school		61 (39.6)	18 (25.4)	
Junior high school		48 (31.2)	24 (33.8)	
Senior high school		42 (27.3)	24 (33.8)	
College/academy		3 (1.9)	2 (2.8)	
Other			3 (4.2)	
Mother				
Age (year) mean (SD)		35.42 (7.1)	30.62 (8.04)	<0.001^*^
Height (cm) mean (SD)		150.75 (5.72)	153.8 (6.83)	0.001^*^
Education, n (%)				0.176^†^
Elementary school		58 (37.7)	12 (25.5)	
Junior high school		56 (36.4)	18 (38.3)	
Senior high school		38 (24.7)	16 (34.0)	
College/academy		1 (0.6)		
Other		1 (0.6)	1 (2.1)	
Age on birth of subjects (year) mean (SD)		27.45 (6.5)	28.15 (7.32)	0.428^*^
Parity, median (min., max.)		2 (1, 6)	2 (1, 5)	0.54^*^
Family members who smoke, n (%)				0.132^*^
Yes		119 (77.3)	40 (85.1)	
No.		35 (22.7)	7 (14.9)	
Family income, n (%)				0.027^*^
<Rp. 2,893,100		130 (84.4)	51 (71.8)	
≥Rp. 2,893,100		24 (15.6)	20 (28.2)	

SD, standard deviation; ^*^Mann-Whitney test, ^†^*chi*-square test. Classification of family income based on the regional minimum wage of Bandung Regency in 2019 (27).

Children with short stature were significantly older than those with normal stature (mean [SD]: 44.6 [10.02] vs. 39.75 [9.73] months, p = 0.001). Birth weight and birth length were significantly lower in the short-stature group (p = 0.022 and p = 0.015, respectively). Exclusive breastfeeding was more frequently reported among children with short stature (p < 0.001). The mean paternal age was lower, while maternal age was higher in the short-stature group (p = 0.001 and p < 0.001, respectively). Both paternal and maternal heights were significantly lower, and family income was also lower in children with short stature (p = 0.002, p = 0.001, and p = 0.022, respectively). No significant differences were observed in sex, immunization history, parental education, maternal age at childbirth, parity, or household smoking exposure.

Biochemical analysis showed that IGF-1, calcium, phosphorus, and magnesium levels were significantly lower in children with short stature compared to those with normal stature (all p < 0.05), whereas no significant difference was found for vitamin 25(OH)D levels (p = 0.059) (**Table 2**).

**Table 2:** Differences in levels of IGF-1, vitamin 25(OH)D, calcium, phosphorus, and magnesium in children aged 24–59 months.

Variable	Short stature	Normal stature	P value
	HAZ <-2 SD (n=154)	2SD ≤ HAZ ≥- 2SD (n=71)	
IGF-1 median (min., max.) (ng/mL)	51 (4, 354)	100 (11, 378)	0.004^*^
Vitamin 25(OH)D (ng/mL) mean (SD)	21.77 (5.10)	23.19 (5.29)	0.059^‡^
Calcium ion (mg/dL) mean (SD)	4.57 (0.69)	4.87 (0.61)	0.004^‡^
Phosphorus (mg/dL) mean (SD)	5.52 (0.96)	5.89 (1.24)	0.026^‡^
Magnesium (mg/dL) mean (SD)	2.24 (0.17)	2.32 (0.22)	0.013^‡^

SD, standard deviation; ^*^Mann-Whitney test, ^‡^t-student test.

To further explore heterogeneity within short stature, subgroup analyses were performed based on weight-for-age status: underweight short stature (n = 84), normal-weight short stature (n = 70), and normal-weight normal stature (n = 71). Comparisons of baseline characteristics are presented in **Table 3**, and biomarker levels are shown in **Table 4**. Children with underweight short stature had significantly lower birth weight and birth length compared to those with normal-weight normal stature (p < 0.05), while no significant differences were observed between the underweight and normal-weight short stature groups. Family income was also lower in the underweight short stature group. Other variables were comparable across groups.

**Table 3:** Comparison of basic subject characteristics based on stature and weight-for-age in children aged 24–59 months.

Variable	Underweight short stature	Normal weight short stature	Normal weight normal stature	P value
	(n=84)	(n=70)	(n=71)	
Age (month) mean (SD)	45 (10.0)	44.19 (10.192)	39.75 (9.73)	0.003^*^
Gender. n (%)				0.862^†^
Male	38 (45.2)	34 (48.6)	35 (49.3)	
Female	46 (54.8)	36 (51.4)	36 (50.7)	
Birth weight (g) mean (SD)	2,715.5 (403.89)	2,842 (551.65)	2,955 (433.25)	0.006^*^
< 2,500 g, n (%)	14 (16.7)	10 (14.3)	8 (11.3)	
≥ 2,500 g, n (%)	70 (83.3)	60 (85.7)	63 (88.7)	
Birth length (cm) mean (SD)	47.79 (2.99)	47.93 (2.48)	48.92 (2.08)	0.016^*^
Exclusive breastfeeding history, n (%)				<0.001^†^
Yes	75 (89.3)	62 (88.6)	45 (63.4)	
No.	9 (10.7)	8 (11.4)	26 (36.6)	
Immunization history, n (%)				0.884^†^
Complete	71 (84.5)	60 (85.7)	62 (87.3)	
Incomplete	13 (15.5)	10 (14.3)	9 (12.7)	
Father				
Age (year) mean (SD)	34.93 (7.27)	36.01 (6.9)	34.35 (7.92)	0.450*
Height (cm) mean (SD)	159.56 (6.29)	161.5 (7.05)	163.31 (6.79)	0.003*
Education, n (%)				0.121†
Elementary school	34 (40.5)	27 (38.6)	18 (25.4)	
Junior high school	21 (25)	27 (38.6)	24 (33.8)	
Senior high school	28 (33.3)	14 (20)	24 (33.8)	
College/academy	1 (1.2)	2 (2.9)	2 (2.8)	
Other	0	0	3 (4.2)	
Mother				
Age (year) mean (SD)	30.88 (6.25)	31.51 (6.87)	30.62 (8.04)	0.740*
Height (cm) mean (SD)	150.19 (4.94)	151.43 (6.5)	153.8 (6.83)	0.001*
Education, n (%)				0.211†
Elementary school	30 (35.7)	28 (40)	12 (25.5)	
Junior high school	28 (33.3)	28 (40)	18 (38.3)	
Senior high school	25 (29.8)	13 (18.6)	16 (34.0)	
College/academy	1 (1.2)	0	0	
Other	0	1 (1.4)	1 (2.1)	
Age at childbirth (year) mean (SD)	27.12 (6.24)	27.86 (6.82)	28.15 (7.32)	0.485*
Parity, median (min., max.)	2.00 (1, 6)	2.00 (1, 6)	2.00 (1, 5)	0.556*
Family members who smoke, n (%)				0.301^†^
Yes	64 (76.2)	55 (78.6)	40 (85.1)	
No	20 (23.8)	15 (21.4)	7 (14.9)	
Family income, n (%)				0.022^†^
<Rp. 2,893,100	75 (89.3)	55 (78.6)	51 (71.8)	
≥Rp. 2,893,100	9 (10.7)	15 (21.4)	20 (28.2)	

SD, standard deviation; ^*^Kruskal-Wallis test, ^†^Chi-square test, Classification of family income based on the regional minimum wage of Bandung Regency in 2019 (27).

**Table 4:** Differences in levels of IGF-1, vitamin 25(OH)D, calcium, phosphorus, and magnesium based on stature and weight-for-age in children aged 24–59 months.

Variable	Underweight short stature (A)	Normal weight short stature (B)	Normal weight normal stature (C)	P value	P value A vs B	P value A vs C	P value B vs C
	(n=84)	(n=70)	(n=71)				
IGF-1 (ng/mL) median (min., max.)	44.5 (4, 301)	72.45 (8, 354)	100 (11, 378)	0.004^*^	0.517^□^	0.035^□^	0.376^□^
Vitamin 25(OH)D (ng/mL), mean (SD)	21.12 (5.25)	22.5 (4.86)	23.19 (5.29)	0.045^‡^	0.232^◼^	0.040^◼^	0.706^◼^
Calcium ion (mg/dL), mean (SD)	4.45 (0.64)	4.69 (0.72)	4.87 (0.61)	0.002^‡^	0.085^◼^	0.001^◼^	0.296^◼^
Phosphor (mg/dL), mean (SD)	5.42 (0.86)	5.63 (1.06)	5.89 (1.24)	0.044^‡^	0.490^◼^	0.033^◼^	0.355^◼^
Magnesium (mg/dL), mean (SD)	2.21 (0.16)	2.28 (0.19)	2.32 (0.22)	0.008^‡^	0.150^◼^	0.006^◼^	0.418^◼^

SD, standard deviation; ^*^Kruskal-Wallis test, ^‡^ANOVA test, ^□^Bonferroni correction, ^◼^Tukey’s HSD test.

Biomarker levels differed significantly across the three groups (all p < 0.05). *Post-hoc* analysis demonstrated that children with normal-weight normal stature had significantly higher levels of IGF-1, vitamin 25(OH)D, calcium, phosphorus, and magnesium compared to those with underweight short stature, whereas no significant differences were observed between the underweight and normal-weight short stature groups or between the normal-weight short stature and normal-weight normal stature groups (**Table 4**).

The prevalence of low IGF-1 levels was highest in the underweight short stature group. Overall, 43 children (19.1%) had low IGF-1 levels (**Table 5**). Hypovitaminosis D was highly prevalent (94.1%), including 28.8% with deficiency and 65.3% with insufficiency, with the highest proportion observed in the underweight short stature group (**Table 6**). Low calcium levels were observed in 45.8% of subjects, while low phosphorus levels were found in 13.3% (**Tables 7**, **8**). Most children had normal magnesium levels, although 12.9% had elevated levels (**Table 9**).

**Table 5:** Classification of IGF-1 levels based on normal ranges in children aged 24–59 months.

IGF-1 levels	Underweight short stature	Normal weight short stature	Normal weight normal stature
	(n=84)	(n=70)	(n=71)
Boy			
Low, n (%)	10 (11.91)	4 (5.71)	5 (7.04)
Normal, n (%)	27 (32.14)	28 (40.00)	28 (39.44)
High, n (%)	1 (1.19)	2 (2.86)	2 (2.82)
Girl			
Low, n (%)	15 (17.86)	4 (5.71)	5 (7.04)
Normal, n (%)	30 (35.71)	31 (44.29)	27 (38.03)
High, n (%)	1 (1.19)	1 (1.43)	4 (5.63)

Classification of IGF-1 levels based on normal ranges. Boy aged two years (16–222 ng/mL), three years (22–229 ng/mL), and four years (30–236 ng/mL). Girl aged two years (28–256 ng/mL), three years (31–249 ng/mL), and four years (33–237 ng/mL) (20).

**Table 6:** Classification of vitamin 25(OH)D levels based on normal ranges in children aged 24–59 months.

Vitamin 25(OH)D	Underweight short stature	Normal weight short stature	Normal weight normal stature
	(n=84)	(n=70)	(n=71)
Deficiency, n (%)	33 (39.3)	16 (22.9)	16 (22.5)
Insufficiency, n (%)	48 (57.1)	51 (72.9)	48 (67.6)
Normal, n (%)	3 (3.6)	3 (4.2)	7 (9.9)

Classification of deficiency (≤20 ng/mL), insufficiency (21–29 ng/mL), normal (30–00 ng/mL) (22).

**Table 7:** Classification of calcium levels based on normal ranges in children aged 24–59 months.

Calcium Levels	Underweight short stature	Normal weight short stature	Normal weight normal stature
	(n=84)	(n=70)	(n=71)
Low, n (%)	38 (45.2)	32 (45.7)	33 (47.1)
Normal, n (%)	29 (34.5)	19 (27.1)	32 (45.7)
High, n (%)	17 (20.2)	19 (27.1)	5 (7.1)

Classification of calcium levels based on the normal range of 4,8–5,2 mg/dL (23).

**Table 8:** Classification of phosphorus levels based on normal ranges in children aged 24–59 months.

Phosphorus levels	Underweight short stature	Normal weight short stature	Normal weight normal stature
	(n=84)	(n=70)	(n=71)
Low, n (%)	19 (22.6)	5 (7.1)	6 (8.5)
Normal, n (%)	65 (77.4)	65 (92.9)	65 (91.5)
High, n (%)	0	0	0

Classification of phosphorus levels based on the normal range of (4,0–7,0 mg/dL) (25).

**Table 9:** Classification of magnesium levels based on normal ranges in children aged 24–59 months.

Magnesium levels	Underweight short stature	Normal weight short stature	Normal weight normal stature
	(n=84)	(n=70)	(n=71)
Low, n (%)	0	0	0
Normal, n (%)	70 (83.3)	58 (82.9)	68 (95.8)
High, n (%)	14 (16.7)	12 (17.1)	3 (4.2)

Classification of magnesium levels based on the normal range of (1,8–2,4 mg/dL) (26).

Multivariate logistic regression analysis showed that IGF-1 levels (OR 0.94, 95% CI 0.90–0.98, p=0.041), also maternal education (OR 0.453, 95% CI 0.261-0.788, p=0.005) were significantly associated with short stature. Higher IGF-1 levels and maternal education were associated with a decreased likelihood. Other variables, including vitamin D levels, and mid-parental height, were not significantly associated with the outcome. (**Table 10**).

**Table 10:** Association of IGF-1, vitamin 25(OH)D, calcium, phosphorus, and magnesium levels with the incidence of short stature in children aged 24–59 months after controlling for confounding variables.

Variable	Adjusted OR	95% CI	p-value
IGF-1	0.994	0.988 – 1.000	0.041
Vitamin D	0.958	0.887 – 1.035	0.275
Calcium	0.318	0.146 – 0.694	0.004
Phosphorus	0.713	0.476 – 1.067	0.100
Magnesium	0.063	0.006 – 0.659	0.021
Age	1.048	1.007 – 1.092	0.021
Birth weight	0.999	0.998 – 1.000	0.294
Birth length	0.855	0.686 – 1.067	0.166
Income	0.538	0.207 – 1.397	0.023
Father’s education	1.461	0.891 – 2.394	0.133
Mother’s education	0.453	0.261 – 0.788	0.005
Father’s height	0.988	0.933 – 1.047	0.693
Mother’s height	1.010	0.947 – 1.078	0.754

data analyzed using logistic regression, OR, Odd Ratio; CI, Confidence Interval.

Further analysis demonstrated significant differences among groups in age, birth weight, birth length, paternal height, and maternal height (all p < 0.05). *Post-hoc* analysis revealed that these differences were primarily observed between the underweight short stature and normal-weight normal stature groups, while no significant differences were found between the other groups (**Table 11**).

**Table 11:** Continuation comparison of basic subject characteristics based on stature and weight-for-age in children aged 24–59 months.

Variable	Underweight short stature (A)	Normal weight short stature (B)	Normal weight normal stature (C)	P value	P value A vs B	P value A vs C	P value B vs C
	(n=84)	(n=70)	(n=71)				
Age (month) mean (SD)	45 (10.0)	44.19 (10.192)	39.75 (9.73)	0.003^*^	0.8^□^	0.003^□^	0.023^□^
Birth weight (g) mean (SD)	2.715.5 (403.89)	2.842 (551.65)	2.955 (433.25)	0.006^*^	0.212^□^	0.004^□^	0.319^□^
Birth length (cm) mean (SD)	47.79 (2.99)	47.93 (2.48)	48.92 (2.08)	0.016^*^	0.942^□^	0.020^□^	0.061^□^
Father’s Age (year) mean (SD)	34.93 (7.27)	36.01 (6.9)	34.35 (7.92)	0.450*	0.675^□^	0.894^□^	0.428^□^
Father’s Height (cm) mean (SD)	159.56 (6.29)	161.5(7.05)	163.31 (6.79)	0.003*	0.171^□^	0.002^□^	0.240^□^
Mother’s Age (year) mean (SD)	30.88 (6.25)	31.51(6.87)	30.62 (8.04)	0.740*	0.844^□^	0.971^□^	0.732^□^
Mother’s Height (cm) mean (SD)	150.19 (4.94)	151.43(6.5)	153.8 (6.83)	0.001*	0.422^□^	0.01^□^	0.56^□^
Mother’s Age at childbirth (year) mean (SD)	27.12 (6.24)	27.86(6.82)	28.15 (7.32)	0.485*	0.780^□^	0.963^□^	0.611^□^
Parity, median (min., max.)	2.00 (1, 6)	2.00 (1, 6)	2.00 (1, 5)	0.556*	0.679^□^	0.687^□^	1^□^

SD, standard deviation; ^*^Kruskal-Wallis test, ^□^Bonferroni correction.

Subgroup multivariate logistic regression analyses (A vs C and B vs C) were performed as a sensitivity analysis and are presented in **Table 12**. The results demonstrated that the associations observed in the primary model were largely driven by the underweight short stature group (A). Several variables, including IGF-1, vitamin D, calcium, phosphorus, and magnesium, were significantly associated with short stature in the A vs C comparison. In contrast, in the B vs C comparison, most variables were not significantly associated with the outcome, except for magnesium and age, suggesting different underlying mechanisms between malnourished and non-malnourished short stature (**Table 12**).

**Table 12:** Multivariate logistic regression analysis including subgroup comparisons (A vs C and B vs C).

Variable	A vs C (OR, 95% CI)	p-value	B vs C (OR, 95% CI)	p-value
IGF-1	0.987 (0.979–0.996)	0.003	0.996 (0.991–1.002)	0.160
Vitamin D	0.850 (0.732–0.987)	0.033	0.971 (0.899–1.048)	0.449
Calcium	0.152 (0.055–0.416)	<0.001	0.611 (0.281–1.327)	0.213
Phosphorus	0.348 (0.175–0.694)	0.003	0.801 (0.542–1.183)	0.265
Magnesium	0.020 (<0.001 –0.955)	0.047	0.105 (0.011–0.982)	0.048
Age	1.094 (1.017–1.176)	0.016	1.050 (1.008–1.093)	0.019
Birth weight	0.997 (0.995–0.999)	0.007	1.000 (0.999–1.001)	0.780
Birth length	1.140 (0.858–1.514)	0.365	0.788 (0.619–1.003)	0.053
Income	0.181 (0.033–0.992)	0.049	0.792 (0.331–1.894)	0.600
Father’s age	0.948 (0.871–1.032)	0.216	1.005 (0.948–1.066)	0.857
Mother’s age	1.066 (0.969–1.171)	0.188	1.005 (0.942–1.073)	0.871
Father’s height	0.959 (0.875–1.050)	0.362	0.992 (0.936–1.052)	0.793
Mother’s height	1.034 (0.924–1.157)	0.559	0.993 (0.931–1.060)	0.843

A, underweight short stature; B, normal weight short stature; C, normal weight normal stature. All models were adjusted for the variables included in **Table 10**.

## 4 Discussion

The primary finding of this study is that children aged 24–59 months with short stature and underweight status exhibit the lowest levels of IGF-1, vitamin 25(OH)D, calcium, phosphorus, and magnesium compared to their normal-stature peers. Furthermore, multivariable analysis identifies IGF-1, calcium, phosphorus, magnesium, family income, and maternal education, as key factors significantly associated with the occurrence of short stature in this population. These findings highlight the critical interplay between socioeconomic status, endocrine regulation, and bone mineral metabolism in early childhood growth.

This study found that children with short stature had significantly lower birth weight and birth length compared with children of normal stature, particularly in the underweight subgroup (**Tables 1**, **3**, **11**). Interestingly, a history of exclusive breastfeeding was more frequently reported among children with short stature than among those with normal stature in this study (**Table 1**). Previous studies have demonstrated that exclusive breastfeeding positively influences weight gain and linear growth in infants younger than six months, thereby reducing the risk of stunting (28). However, Yilak et al. (29) reported that ineffective breastfeeding practices such as improper positioning, poor latch, and suboptimal suckling technique may limit the benefits of exclusive breastfeeding by reducing breast milk intake, potentially leading to inadequate weight gain, stunting, and increased susceptibility to infections.

Family income was significantly lower in the short-stature group compared with the normal-stature group, with the lowest income observed among children with underweight short stature compared with those with normal-weight short stature and normal-weight normal stature (**Tables 1**, **3**). Multivariable logistic regression analysis examining the association between biological marker levels and short stature, after adjustment for potential confounders, demonstrated that family income was independently associated with the occurrence of short stature (**Tabls 10**, **12**). Adequate socioeconomic conditions are known to positively influence multiple aspects of children’s well-being, including health, education, environmental exposure, and social development (30).

Significant differences in parental age were observed when comparing the short-stature and normal-stature groups: fathers were younger and mothers were older in the short-stature group (**Table 1**). However, after stratification into three groups (underweight short stature, normal-weight short stature, and normal-weight normal stature), no significant differences in parental age were found among the groups (**Tables 3**, **11**). Younger paternal age has been associated with an increased risk of low birth weight (31), whereas advanced maternal age has been linked to a higher risk of preterm birth and low birth weight in offspring (32).

This study found that immunization status did not differ significantly between groups, with most children having complete immunization coverage. Nevertheless, immunization plays a critical role in preventing infectious diseases that may impair growth. Children who do not receive complete immunization have been reported to have a 1.47-fold higher risk of short stature compared with fully immunized children (33).

No significant differences were observed among the groups with respect to paternal and maternal education level, parity, or the presence of household members who smoke. However, multivariable logistic regression analysis assessing the association between biological marker levels and short stature, after adjustment for potential confounders, demonstrated that maternal education level was independently associated with short stature (**Table 10**). This finding is consistent with previous studies showing that lower maternal education may influence child growth through limited health literacy, suboptimal childcare practices, and reduced access to healthcare resources (34).

Parental height was lower in children with underweight short stature in the baseline analysis, suggesting a role of genetic factors in growth (**Tables 1**, **3**). However, this association was not significant in the multivariate model, indicating that its effect may be attenuated after adjusting for nutritional and biochemical factors (**Tables 11**, **12**). These findings suggest that, although genetic potential contributes to growth, its influence may be less prominent than modifiable factors, particularly in undernourished children. Genetic contributions to height increase with age, growth outcomes in children are strongly influenced by inherited factors, alongside modifiable environmental determinants (35).

Endocrine biomarkers may be influenced by external factors, including seasonal variation, as hormone levels such as growth hormone can fluctuate across environmental conditions (36). In addition, seasonal patterns in growth and IGF-1 levels have been observed in children, with peak linear growth typically occurring during spring (37). In this study, sample collection was not standardized by season, which may have introduced variability in biomarker measurements. However, despite this potential variability, IGF-1 levels remained significantly lower in the short-stature group compared with the normal-stature group (**Table 2**), suggesting that the observed association is robust. When stratified into three groups, children with underweight short stature had the lower IGF-1 levels compared with those with normal-weight normal stature (**Table 4**). The highest proportion of low IGF-1 levels was observed in the underweight short stature group, with 29.77% of subjects classified as having low IGF-1 (**Table 5**). The significantly lower IGF-1 levels observed in the short stature group in this study are consistent with recent findings by Senudin et al (14),. IGF-1 is a growth-promoting hormone primarily regulated by growth hormone (GH) and plays a central role in cell proliferation, anabolic processes, and bone formation. Nutritional deficiencies, particularly inadequate energy and protein intake, may reduce IGF-1 levels through increased protein catabolism and GH resistance, thereby contributing to impaired linear growth (38).

In this study, mean (SD) serum 25-hydroxyvitamin D [25(OH)D] levels did not differ significantly between the short-stature and normal-stature groups (**Table 2**). However, after stratification, children with underweight short stature had significantly lower 25(OH)D levels compared with those with normal-weight normal stature (**Table 4**). Our findings concordant with a global meta-analysis that short stature children not only exhibit lower serum 25(OH)D levels but also face a 1.13-fold higher risk of vitamin D deficiency compared to children with normal stature (15). Hypovitaminosis D was highly prevalent, affecting 94.1% of subjects, with 65.3% classified as insufficient and 28.8% as deficient. Vitamin D deficiency was most common in the underweight short stature group (**Table 6**). These findings are supported by Mokhtar et al, who reported lower serum 25(OH)D levels in short stature children compared to normal stature peers (16). According to the Institute of Medicine (IOM), vitamin 25(OH)D serum levels ≥20 ng/mL are considered sufficient for bone health in children (39). Underweight toddlers have an increased risk of vitamin D deficiency, which may impair calcium and phosphorus homeostasis and disrupt bone mineralization (16, 40).

Calcium levels were significantly lower in children with short stature, particularly in those with underweight short stature, although the prevalence of hypocalcemia was similar across groups (**Tables 2**, **4**, **7**). Overall, nearly half of the participants had low calcium levels, with hypovitaminosis D commonly observed in the underweight short stature group. Adequate calcium and vitamin D are essential for optimal bone mineralization and linear growth (41). Phosphorus levels were significantly lower in children with short stature, particularly in those with underweight short stature, with the highest prevalence of low phosphorus levels observed in this group (**Tables 2**, **4**, **8**). This may be partly explained by the high prevalence of hypovitaminosis D, which can impair calcium and phosphorus absorption (41). Similarly, magnesium levels were lower in children with short stature and lowest in those with underweight short stature, although most values remained within the normal range (**Tables 2**, **4**, **9**). Given the role of magnesium in vitamin D activation and parathyroid hormone regulation, deficiency may further disrupt calcium homeostasis and bone mineralization (41, 42).

The biochemical analysis revealed significantly lower levels of IGF-1, vitamin D, calcium, phosphorus, and magnesium in the underweight short stature group compared with children with normal growth, while no significant differences were observed between normal-weight short stature and normal-weight normal stature (**Table 4**). The multivariate analysis further supports this interpretation, demonstrating that the associations between short stature and multiple biomarkers were largely driven by the underweight subgroup (**Table 12**). In these children, reduced IGF-1 levels may reflect impairment of the GH–IGF-1 axis, while deficiencies in calcium, phosphorus, and magnesium indicate suboptimal mineral availability for bone growth (43).

No significant differences were observed between children with normal-weight short stature and those with normal-weight normal stature. This finding suggests that, in the absence of undernutrition, growth impairment may be primarily related to intrinsic factors such as genetic potential or constitutional growth patterns rather than nutritional or biochemical disturbances. Although parental height was evaluated, it was not independently associated with short stature in the multivariate model, indicating that its effect may be attenuated after accounting for other factors. These findings challenge the common assumption that short stature is predominantly driven by nutritional deficiency and instead support the concept that short stature is a heterogeneous condition influenced by multiple factors, including genetic predisposition and growth regulatory mechanisms beyond nutritional status (6, 30, 35). This highlights the importance of careful clinical evaluation and avoiding over-attribution of short stature to nutritional causes, particularly in children with adequate weight.

Our findings partially support our hypothesis. Significant differences in serum IGF-1, 25-hydroxyvitamin D, calcium, phosphorus, and magnesium were observed across children with underweight short stature, normal-weight short stature, and normal-weight normal stature; however, *post-hoc* analysis and multivariate logistic regression analysis including subgroup comparisons showed that these differences were observed only between children with underweight short stature and those with normal-weight normal stature, while no significant differences were found between the other groups.

## 5 Conclusion

Reduced levels of IGF-1, calcium, phosphorus, and magnesium are associated with short stature in children aged 24–59 months. These associations are predominantly observed in children with concurrent undernutrition, suggesting that disturbances in endocrine and mineral homeostasis contribute to impaired linear growth primarily in this subgroup. This finding underscores that short stature is a heterogeneous condition and highlights the importance of integrating nutritional and endocrine evaluation in its clinical assessment.

## 6 Strengths and limitations

The strengths of this study are:

1. The study evaluated comprehensive growth-related biomarkers, including IGF-1 and key components of bone mineral metabolism (25-hydroxyvitamin D, calcium, phosphorus, and magnesium).
2. The stratification of participants into underweight short stature and normal-weight short stature provided clinically relevant differentiation and enabled a clearer interpretation of the role of nutritional status in growth impairment.
3. Anthropometric assessment was conducted by trained pediatrician using standardized procedures and classified according to the WHO Child Growth Standards 2006, ensuring methodological consistency and comparability with international data.
4. Serum samples were stored at −80 °C prior to analysis, which helps maintain biomarker stability and supports the reliability of laboratory measurements.
5. Multivariable analysis was performed to control for potential confounding factors, strengthening the validity of the observed associations.
6. Additional *post-hoc* and multivariate logistic regression analysis enabled identification of specific group differences, revealing that biochemical alterations were confined to children with underweight short stature, thereby highlighting the heterogeneity of short stature.

The limitations of this study are:

1. The cross-sectional design precludes causal inference between biomarkers and short stature.
2. Recruitment through primary healthcare facilities may have introduced selection bias.
3. The single-district setting limits the generalizability of the findings to other populations.
4. Detailed dietary intake was not assessed, limiting control for nutritional confounders.
5. The use of total sampling without an *a priori* sample size calculation may have limited statistical power and increased the risk of type II error; additionally, biomarkers were measured at a single time point and may not reflect long-term status.

## Data Availability

The original contributions presented in the study are included in the article/supplementary material. Further inquiries can be directed to the corresponding author.

## Glossary

Ca: calcium
CDC: Centers for Disease Control and Prevention
Direct-ISE: direct ion selective electrode
GH: growth hormone
GHRH: growth hormone-releasing hormone
IDAI: Indonesian Pediatric Association
IGF-1: insulin-like growth factor 1
MTB: methyl thymol blue
Mg: magnesium
Ph: phosphorus
PTH: parathyroid hormone
SBS: stored biological specimens
SD: standard deviation
WHOCGS: World Health Organization child growth standards
